# Case Report: Endoscopic removal of a giant complex gastric trichobezoar: an alternative strategy using combined techniques

**DOI:** 10.3389/fmed.2026.1950339

**Published:** 2026-10-01

**Authors:** Hai-Hua Zhou, Jian-Ping Xu, Rui-Qiang Li, Wang Miao, Jin-Jin Xie, Wei Zhang, Min Zhou, Shuai Yuan

**Affiliations:** 1 Department of Endoscopy Center, Xiaolan Clinical Institute of Shantou University Medical College, Zhongshan, China; 2 Department of Oncology, Xiaolan Clinical Institute of Shantou University Medical College, Zhongshan, China; 3 Department of Pediatrics, Xiaolan Clinical Institute of Shantou University Medical College, Zhongshan, China; 4 Department of Gastroenterology, Xiaolan Clinical Institute of Shantou University Medical College, Zhongshan, China

**Keywords:** case report, endoscopic removal, gastric trichobezoar, high-power electrocoagulation, minimally invasive therapy, pediatric patient

## Abstract

**Background:**

Giant gastric trichobezoar is a rare foreign body resulting from chronic hair ingestion and may lead to serious complications, including obstruction, perforation, and malnutrition. Conventional endoscopic removal often fails due to its large size, smooth, elastic surface, and tensile strength. Therefore, surgery is often the standard treatment. In this report, we discuss the case of a girl with a giant trichobezoar containing cotton threads, which was successfully removed following forceps-assisted mechanical fragmentation and transparent cap-assisted extraction.

**Case summary:**

A 7-year-old girl with a 5-year history of trichophagia presented with abdominal pain, distension, palpable mass, and iron-deficiency anemia. Imaging and endoscopy revealed a 12 cm × 6 cm gastric trichobezoar. As surgery was declined, endoscopic removal under general anesthesia was performed. The procedure used high-power electrocoagulation (EndoCut/Forced Coag mode) to segment the dense bezoar, followed by piecemeal extraction with forceps and cap-assisted removal to prevent cardia laceration and esophageal injury. The trichobezoar was successfully removed without mucosal injury, and the patient was discharged 4 days after the endoscopic procedure with complete resolution of symptoms.

**Conclusions:**

With strict patient selection, multidisciplinary teamwork, and adequate expertise and equipment, endoscopic treatment may offer a minimally invasive alternative to the surgical removal of giant gastric trichobezoars in selected pediatric cases. Psychological support is also important to address underlying behavioral factors that may lead to recurrence.

## 1 Introduction

Trichobezoar is a rare foreign body formed by the gradual accumulation of ingested hair in the stomach. It most commonly affects young women with underlying psychiatric disorders such as trichotillomania and trichophagia. The onset typically occurs between 9 and 13 years of age, with an estimated population prevalence of 0.5%–2.0% (1). Over time, hair mixes with mucus and food debris to form a dense, resilient mass, which can cause abdominal pain, early satiety, gastric ulceration, bleeding, obstruction, and even perforation (2). When the trichobezoar extends beyond the stomach into the small intestine, it is referred to as Rapunzel syndrome (3).

For large trichobezoars, open or laparoscopic gastrotomy remains the mainstay of treatment, accounting for approximately 65% and 22% of reported cases, respectively. In contrast, successful endoscopic removal has been reported in only about 13.8% of cases (4). The endoscopic removal of trichobezoars exceeding 10 cm in diameter remains challenging as it often occupies most of the gastric lumen, thus limiting visualization and working space. These lesions also have a smooth, mucus-coated surface that makes them difficult to grasp with forceps. In addition, the densely packed keratinized hair has a high tensile strength and can not easily be destroyed with thermal ablation.

Several endoscopic techniques have been proposed to remove trichobezoars, including fragmentation with argon plasma coagulation, thermal cutting with a hook knife, FlushKnife dissection via a dual-channel endoscope, and cola dissolution. However, the failure rates of these endoscopic removal techniques remain high, particularly for large trichobezoars with resistant components such as cotton threads (5).

In this report, we discuss the case of a 7-year-old girl with a 5-year history of trichophagia who presented with a 12 cm × 6 cm gastric trichobezoar containing abundant cotton threads and food debris. After the family declined surgical gastrotomy, we employed an integrated endoscopic strategy combining high-power endoscopic submucosal dissection (ESD)-based electrocoagulation for segmentation with mechanical fragmentation with foreign-body forceps, and transparent cap-assisted extraction. The trichobezoar was completely and successfully removed without complications.

## 2 Case description

### 2.1 Patient presentation

A 7-year-old girl presented with a one-week history of dull epigastric pain and postprandial fullness without nausea or vomiting. Although her appetite was poor, she had normal bowel habits. Physical examination showed fatigue, pale conjunctivae and lips, asymmetric abdominal distension, and a firm, non-tender, well-demarcated epigastric mass measuring approximately 12 cm × 6 cm with limited mobility. No signs of peritoneal irritation were noted.

The patient had a history of trichophagia beginning at 2 years of age. The behavior was reportedly interrupted when discovered, and the family believed that she had completely stopped in recent years. However, no formal psychiatric intervention or follow-up had been undertaken to confirm the change in behavior.

### 2.2 Diagnostic work-up

Laboratory findings revealed iron-deficiency anemia (hemoglobin 77 g/L, hematocrit 0.267 L/L, serum iron 3.1 μmol/L, ferritin 6.3 ng/mL) and hypoproteinemia (total protein 53.2 g/L, albumin 33.5 g/L, prealbumin 88.6 mg/L). The fecal occult blood test was weakly positive.

Contrast-enhanced abdominal computed tomography (CT) demonstrated a heterogeneous intragastric mass measuring approximately 6 cm × 12 cm, suspicious for a foreign body or trichobezoar, without evidence of extension beyond the stomach (Figure 1C).

**Figure 1:**
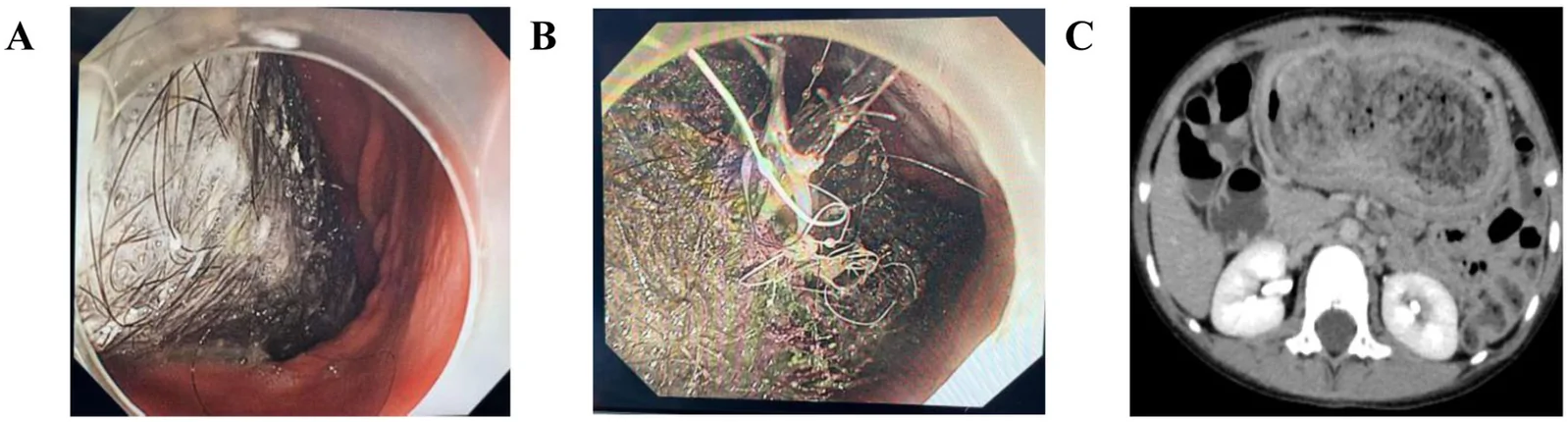
Endoscopic findings of the trichobezoar. **(A)** A large cylindrical trichobezoar extending from the gastric fundus to the antrum (12 cm × 6 cm). **(B)** Dense hair intertwined with abundant cotton threads and food debris. **(C)** Abdominal CT showing a heterogeneous intragastric mass with mottled densities.

Upper endoscopy revealed a large cylindrical trichobezoar extending from the gastric fundus to the antrum, measuring approximately 12 cm × 6 cm (Figure 1A). The bezoar was composed of densely packed hair, abundant cotton threads, and food debris, with a thick mucus coating, a firm consistency, and elastic resilience (Figure 1B). Focal areas of erosion and ulceration were observed in the gastric mucosa.

Following multidisciplinary discussion involving a pediatrician, gastrointestinal surgeon, and gastroenterologist, the patient's family was counselled regarding the available treatment options, including the advantages, limitations, and potential risks of each approach. Laparoscopic or open gastrotomy was expected to require only 1 to 2 h of anesthesia. However, this procedure carries risks including wound infection, visible scarring, contamination of the peritoneal cavity with gastric contents, postoperative adhesions, and a longer recovery period. Endoscopic removal was considered technically feasible because the trichobezoar was accessible, adequate intragastric working space was available, and there was no evidence of intestinal extension, acute obstruction, or perforation. The family was informed that endoscopic treatment was not a first-line alternative to laparoscopy and could require approximately 5 h or longer, with surgical conversion available if necessary. The team established predefined criteria for termination of the endoscopic procedure and conversion to surgery before the intervention. These included overt gastric perforation or uncontrolled active bleeding, failure to achieve a meaningful reduction in bezoar size after 2 h of continuous endoscopic manipulation, severe hemodynamic instability or uncorrectable electrolyte disturbances during anesthesia, and suspected distal migration of fragmented bezoar material or small-bowel obstruction. If any of these criteria were met, the endoscopic procedure would be terminated and surgical management undertaken. We defined these criteria empirically based on our institutional experience and the specific clinical characteristics of this case. Given their strong preference to avoid an abdominal incision in their 7-year-old child, the family elected to proceed with the endoscopic approach under this predefined rescue strategy.

### 2.3 Endoscopic procedure

The procedure was performed under general anesthesia with endotracheal intubation using a standard therapeutic gastroscope (Olympus, Tokyo, Japan). Throughout the procedure, the core body temperature was maintained at 36.2–36.8°C using a forced-air warming blanket and fluid warmer, while lung-protective ventilation with low tidal volumes and positive end-expiratory pressure (PEEP) was employed. Arterial blood gas analysis was performed every 2 h to guide electrolyte management, and fluid administration was adjusted according to urine output and central venous pressure (CVP). Passive limb mobilization and minor positional adjustments were also performed every 2 h with the assistance of the anesthesiologist.

Initially, we attempted to remove the trichobezoar using conventional techniques. However, with the foreign-body forceps, the endoscopist could only grasp a few hairs at a time, resulting in extremely low efficiency (Figure 2A). The snare was unable to effectively capture and retrieve the bezoar because of its hard and smooth surface. The marked density of the trichobezoar rendered standard endoscopic foreign-body retrieval methods ineffective.

**Figure 2:**
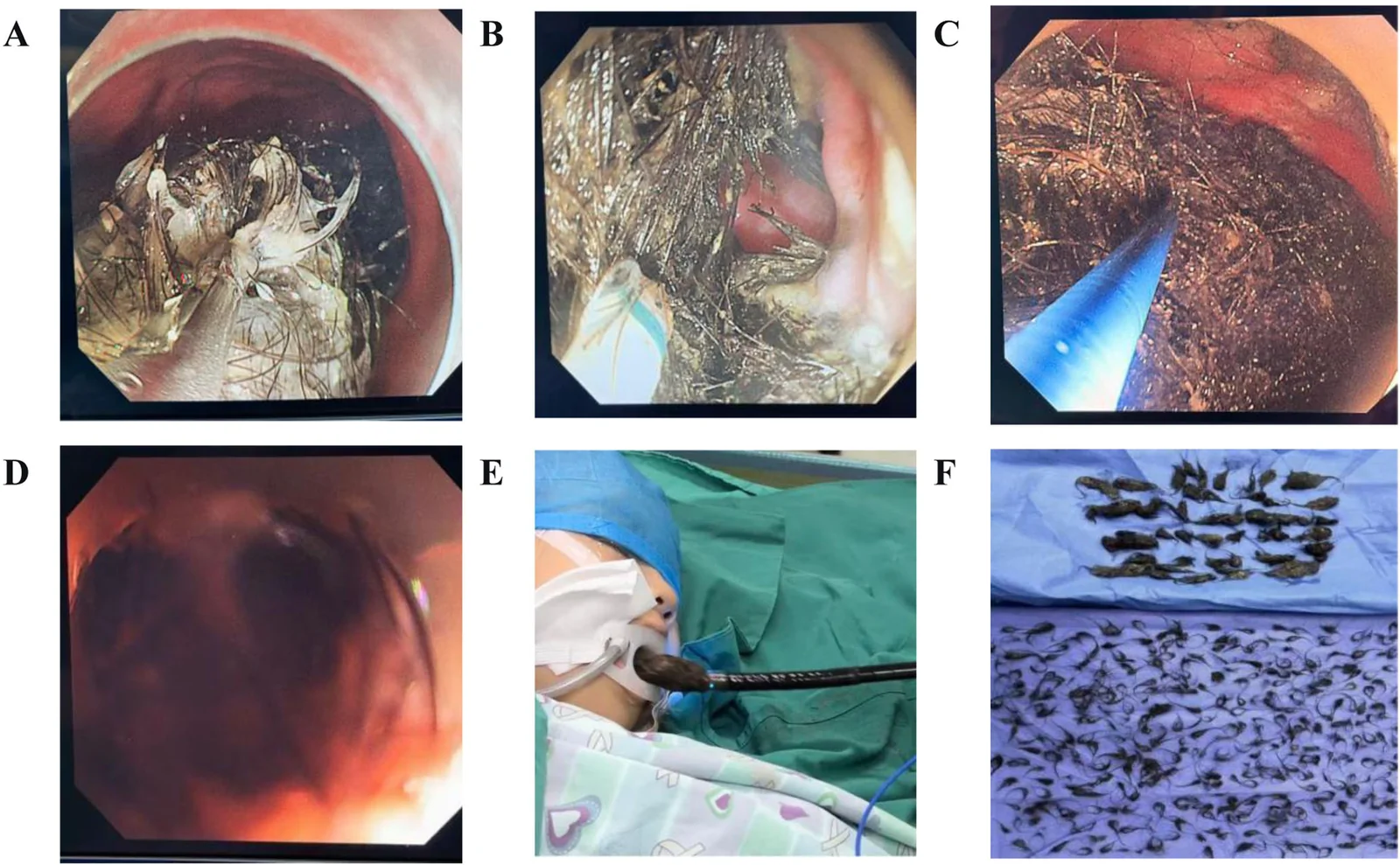
Stepwise endoscopic removal of the trichobezoar. **(A)** Only a few hairs could be grasped with the foreign body forceps. **(B)** The electrosurgical knife operating in high-power hybrid mode (Erbe VIO3, Effect 3, 80 W) segments the bezoar and effectively cuts the cotton fibers. **(C)** The foreign body forceps grasp and tear the loosened trichobezoar. **(D)** A fragment is pulled into a transparent cap mounted on the endoscope tip. **(E)** The endoscope, transparent cap, foreign body forceps, and the grasped trichobezoar fragment are withdrawn together as one unit. **(F)** The extracted trichobezoar.

Based on the physical property that hair can be thermally degraded, we attempted to segment the bezoar using a snare combined with electrosurgical cutting. However, the snare could only engage a very small portion of the trichobezoar, and direct electrocoagulation was inefficient. In particular, the abundant cotton threads interwoven within the trichobezoar exhibited marked resistance to thermal injury and could not be transected.

To overcome these challenges, we used an electrosurgical knife (Micro Tech, Nanjing, China) in a hybrid ESD-derived electrocoagulation cutting mode (Erbe VIO3, Endo Cut effect 3, Forced Coag effect 3, maximum power 80 W). We made the initial approach at the most accessible protuberance of the bezoar (greater curvature side) (Figure 2B, Supplementary Video S1), orienting the knife tip at a 30–45° tangential angle to the bezoar surface. We then manipulated the knife in a tangential shaving manner, analogous to peeling fruit or planing wood, using a superficial reciprocating sliding motion. When the knife was distant from the gastric mucosa, we applied a “downward pressure” cutting strategy to enhance efficiency. Conversely, when the knife approached the mucosa, we switched to a “lifting tension” (or “pull cut”) maneuver, gently elevating the knife tip to keep the cutting edge away from the gastric wall, allowing fine dissection using only the lateral margin or the wire of the knife. We performed intermittent saline instillation to create a fluid cushion separating the bezoar from the gastric wall, thereby providing both visual clarity and a safety margin. After repositioning to expose a new surface, we continued the same shaving strategy layer-by-layer. We extracted fragments immediately after cutting to prevent reaggregation while avoiding rough or forceful traction to prevent mucosal injury.

### 2.4 Fragmentation and extraction

After each incision, the loosened portion was grasped and torn with forceps (Figure 2C) and then pulled into the transparent cap (model D-201-11804) attached to the tip of the endoscope (Figure 2D). The endoscope, cap, forceps, and captured trichobezoar fragment were then withdrawn together as a single unit (Figure 2E).

### 2.5 Operative findings

During the incision, large amounts of dense hair, cotton threads, food debris, and mucus were observed. After complete piecemeal extraction, the gastroscopy reinspection showed no significant gastric mucosal injury, cardia laceration, or esophageal abrasion.

### 2.6 Postoperative course

Given the prolonged procedure duration and the patient's underlying malnutrition, intravenous cefuroxime (50 mg/kg) was administered 30 min before the procedure for antimicrobial prophylaxis and readministered every 8 h postoperatively for a total of 24 h. The patient was also provided with nutritional support and psychological counseling. Postoperatively, the patient developed a transient, mild sore throat, which resolved spontaneously. Endoscopic examination revealed mucosal erosion and ulceration at the site of bezoar impaction. Given that early oral intake might exacerbate local irritation or delay mucosal healing, fasting was maintained for 48 h.

Subsequently, a stepwise oral feeding regimen was initiated, progressing from clear liquids to semi-liquids and then to a soft diet. The patient tolerated the regimen well, without abdominal pain or distension, and was discharged 4 days after the endoscopic procedure. Although the final procedure duration of approximately 15 h substantially exceeded the anticipated time, the family remained satisfied with the outcome, particularly the avoidance of an abdominal incision and the uncomplicated postoperative recovery. Short-term follow-up at 8 weeks showed complete resolution of clinical symptoms and improved appetite. Psychiatric evaluation was arranged to address the underlying trichophagia and prevent recurrence. For the diagnostic and therapeutic procedures, see timeline (Table 1).

**Table 1:** Timeline.

Date	Event
2 years old	Trichophagia was reportedly stopped by the parents, but no formal medical or psychiatric intervention was provided.
March 30, 2026	Patient admitted to the hospital.
April 3, 2026	Underwent endoscopic procedure under general anesthesia with endotracheal Intubation
Postoperative day 1–2	Fasting, prophylactic antibiotics, nutritional support, and intravenous proton pump inhibitor therapy; no bleeding or perforation observed.
Postoperative day 3	Resumed liquid diet, followed by gradual transition to a regular diet; no discomfort reported.
8 weeks after surgery	Follow-up: patient in good condition, with no abdominal symptoms or complications.

## 3 Discussion

Giant gastric trichobezoars are extremely rare and clinically challenging. In this report, we describe the case of a 7-year-old girl who presented with a 5-year history of trichophagia. Subsequently, she developed a 12 cm × 6 cm large intragastric mass composed of densely packed hair, abundant cotton threads, and food debris. Since hair constituted the predominant component and primary structural framework of the intragastric mass, it was classified as a trichobezoar (6, 7). After the family declined surgical gastrotomy, we successfully removed the bezoar using an integrated endoscopic strategy combining high-power ESD-based electrocoagulation for segmentation, mechanical fragmentation with foreign-body forceps, and transparent cap-assisted extraction.

Surgery remains the mainstream treatment for giant trichobezoars. Open or laparoscopic gastrotomy offers direct access, complete removal, and reliable control of potential complications (e.g., gastric perforation). Transumbilical single-incision laparoscopic-assisted gastrotomy is a validated minimally invasive option for pediatric gastric trichobezoars, with reported median operative time of 120 min and hospital stay of 3 days. However, it should be noted that this technique still requires incision of the gastric wall and carries inherent risks of wound infection, abdominal contamination, and adhesions (8). With the increasing availability of advanced endoscopic techniques, minimally invasive endoscopic alternatives have gained attention, particularly for patients who strongly desire minimal trauma and rapid recovery.

Compared with previously reported endoscopic methods, our integrated strategy has several distinct advantages. Early case reports described fragmentation using a polypectomy snare alone. However, this approach was often ineffective for large, resilient bezoars (9, 10). A subsequent report by Wei et al. described endoscopic fragmentation of a trichobezoar in a 5-year-old child using a hook knife. The trichobezoar was divided by thermally induced cutting with Endocut Q mode at 60 W (11).

Pandey et al. described FlushKnife dissection through a dual-channel endoscope, using two alligator forceps to provide countertraction in a 10-year-old child (12). Another report described the use of argon plasma coagulation combined with cola injection (13). However, none of these reports addressed trichobezoars containing abundant cotton threads, which are highly resistant to both thermal breakdown with an electrosurgical cutting device and mechanical fragmentation.

In the present case, lower power settings (40–60 W) and pure cut mode were ineffective against the cotton fibers and yielded extremely low resection efficiency. We therefore switched to a hybrid EndoCut/Forced Coag configuration at 80 W (Effect 3). This choice was based on our observation that this setting was effective in fragmenting the trichobezoar containing dense cotton fibers. We hypothesize that the high peak voltage and low duty cycle characteristic of the Forced Coag mode may have contributed to thermal disruption of the compact fiber architecture. However, the precise mechanism by which electrosurgical energy interacts with non-viable keratinized hair and cotton fibers remains to be elucidated. The coagulation component of the hybrid mode may also promote thermal denaturation of organic debris, thereby facilitating fragmentation (14). The 80 W setting provided sufficient instantaneous energy density even during intermittent current delivery (15, 16). The procedure was guided by tactile feedback. When excessive resistance was encountered during traction, electrosurgical cutting was first used to further fragment the bezoar before traction was resumed. This stepwise approach was more efficient than grasping with foreign body forceps alone.

After segmentation, the atraumatic extraction of fragments is equally critical. In pediatric patients, the risk of cardia laceration or esophageal injury significantly increases when the short axis of a retrieved foreign body exceeds 1.5 cm (17). The majority of the bezoar fragments in our case exceeded this threshold. To address this issue, we introduced transparent cap-assisted extraction, in which each fragment was pulled into the cap before withdrawing the endoscope. Although this technique applies the protective principle of a sheath and is commonly used in foreign body retrieval, it has never been used to remove a trichobezoar. The advantage of this technique is that by enclosing the fragment within the transparent cap during withdrawal, it shields the esophagogastric junction and esophageal mucosa from direct contact with sharp or bulky fragment edges.

Published reports of endoscopic removal of similarly sized gastric bezoars have described procedure durations of approximately 5 h, particularly for phytobezoars, which are generally more brittle and easier to fragment (18). Literature describing endoscopic removal times for trichobezoars remains limited (13). In contrast to previously reported cases, the trichobezoar in our patient was unusually dense and compact, with tightly interwoven cotton fibers that were highly resistant to conventional mechanical and electrosurgical fragmentation. The non-conductive nature of the cotton fibers further reduced the effectiveness of snare electrocoagulation. Overall, the procedure lasted 15 h. During the initial 8 h, forceps grasping and snare electrocoagulation had limited efficacy. After recognizing this limitation, we modified the strategy during the subsequent 6 h by using an electrosurgical knife to create deep furrows and divide the bezoar into smaller segments before piecemeal retrieval. This substantially improved fragmentation efficiency and ultimately enabled complete removal.

However, we acknowledge that, from an anesthetic safety perspective, the prolonged 15 h procedure carried potential risks, including ventilation-associated complications, ventilator-associated pneumonia, hypothermia, fluid and electrolyte disturbances, pressure injuries, and cumulative anesthetic effects. Although in this case, these risks were carefully managed by an experienced anesthesia team through continuous arterial blood gas monitoring, maintenance of core temperature using forced-air warming blankets and fluid warmers, regular positional adjustments, lung-protective ventilation, and pressure-injury prevention, we recognize that such measures can mitigate but not eliminate the risks associated with prolonged anesthesia.

The patient was extubated promptly after the procedure and recovered uneventfully without major complications. Despite the prolonged procedure duration, a single-session approach was preferred to avoid the cumulative risks of repeated anesthesia and intubation, as well as potential mucosal injury and distal migration of fragmented bezoar material between staged procedures. To minimize operator fatigue, two experienced endoscopists alternated every 2–3 h. We acknowledge that the prolonged duration limits the generalizability of this technique. However, the lessons learned from this case may facilitate earlier adoption of the incision-and-segmentation strategy, thereby improving efficiency and reducing procedure time in future similar cases.

Although no procedure-related complications occurred, the technical complexity of this approach means that several precautions are necessary to mitigate the risk of complications, including perforation, bleeding, aspiration, cardia or esophageal injury, postoperative obstruction, and delayed mucosal injury. To reduce the risk of perforation, the knife should be maintained at a tangential angle of approximately 30°–45° to the bezoar surface. Normal saline must be instilled intermittently to create a protective fluid cushion between the bezoar and the gastric wall. Prolonged electrocoagulation at a single site should also be avoided, and the knife tip position must be monitored in real time throughout the procedure. Bleeding risk may be minimized through the use of soft coagulation for immediate hemostasis, with hemostatic clips and diluted epinephrine (1:10,000) available if needed, while avoiding blind grasping and excessive mucosal traction. Aspiration prevention includes the use of a cuffed endotracheal tube, left lateral positioning, intermittent suction of gastric contents through the endoscope working channel, and continuous anesthetic monitoring. Cardia and esophageal injury may be reduced by limiting retrieved fragments to less than 2 cm, minimizing traction, and using a transparent distal cap to protect the mucosa during repeated endoscope passage. To reduce the risk of small-bowel obstruction from distal fragment migration, all visible fragments should be thoroughly retrieved, with close postoperative monitoring for abdominal symptoms and imaging performed when clinically indicated. In addition, to prevent delayed gastric mucosal injury, proton pump inhibitor therapy and early follow-up endoscopy should be performed to assess mucosal healing.

Currently, there is no consensus on the optimal approach to trichobezoar removal. Treatment depends on the size, location, composition, risk of complications, and patient preference, and may involve medical, endoscopic, or surgical management (1, 19). Although our endoscopic approach shows promising potential, its generalizability remains limited by the single-case nature of this report, the need for careful patient selection, and the requirement for specialized expertise and resources. Based on our institutional experience and available literature, this approach should be considered for hemodynamically stable pediatric patients with bezoars confined to the stomach, adequate gastric space for visualization and manipulation, and no evidence of perforation, peritonitis, or intestinal extension, particularly when the family declines surgical intervention. Intestinal extension, extensive synthetic fiber content resistant to fragmentation, severe malnutrition, or contraindications to prolonged anesthesia may favor surgical management. Nevertheless, this technique is resource-intensive and requires specialized equipment, advanced endoscopic skills, multidisciplinary support, and immediate availability of surgical conversion if required. Accordingly, it should be used only in highly selected cases at experienced tertiary-care centers.

Finally, endoscopic removal addresses only the mechanical problem of the bezoar, not the underlying psychiatric disorder of trichophagia. Without appropriate psychological and psychiatric follow-up, recurrence rates are high (20). Long-term follow-up is therefore essential to monitor for recurrent symptoms and ensure ongoing behavioral management. The patient was referred to a psychiatric clinic for formal evaluation after discharge. However, as of the time of this report, the assessment had not yet been completed. Therefore, this case cannot confirm whether the underlying behavioral disorder (trichophagia) has been adequately addressed, nor can it assess the risk of recurrence. We acknowledge that the lack of long-term follow-up is an important limitation of the present report.

## 4 Conclusion

In summary, we report an endoscopic strategy combining high-power ESD-based electrocoagulation, mechanical fragmentation with foreign-body forceps, and transparent cap-assisted extraction that successfully removed a giant complex trichobezoar containing highly resistant cotton fibers in this single pediatric case. Our experience suggests that, in carefully selected pediatric patients whose families decline surgery, this approach may be a technically feasible alternative to open gastrotomy, provided that advanced endoscopic expertise and appropriate psychiatric follow-up are ensured. Nevertheless, the short follow-up period (8 weeks) and the prolonged procedure duration limit the conclusions that can be drawn regarding general safety, efficacy, and recurrence prevention.

## Data Availability

The original contributions presented in the study are included in the article/Supplementary Material, further inquiries can be directed to the corresponding author/s.
